# Using a new artificial intelligence‐aided method to assess body composition CT segmentation in colorectal cancer patients

**DOI:** 10.1002/jmrs.798

**Published:** 2024-05-22

**Authors:** Ke Cao, Josephine Yeung, Yasser Arafat, Jing Qiao, Richard Gartrell, Mobin Master, Justin M. C. Yeung, Paul N. Baird

**Affiliations:** ^1^ Department of Surgery, Western Precinct University of Melbourne Melbourne Victoria Australia; ^2^ Department of Colorectal Surgery Western Health Melbourne Victoria Australia; ^3^ Department of Radiology Western Health Melbourne Victoria Australia; ^4^ Department of Surgery University of Melbourne Melbourne Victoria Australia

**Keywords:** Artificial intelligence, automated segmentation, body composition, colorectal cancer, computed tomography

## Abstract

**Introduction:**

This study aimed to evaluate the accuracy of our own artificial intelligence (AI)‐generated model to assess automated segmentation and quantification of body composition‐derived computed tomography (CT) slices from the lumber (L3) region in colorectal cancer (CRC) patients.

**Methods:**

A total of 541 axial CT slices at the L3 vertebra were retrospectively collected from 319 patients with CRC diagnosed during 2012–2019 at a single Australian tertiary institution, Western Health in Melbourne. A two‐dimensional U‐Net convolutional network was trained on 338 slices to segment muscle, visceral adipose tissue (VAT) and subcutaneous adipose tissue (SAT). Manual reading of these same slices of muscle, VAT and SAT was created to serve as ground truth data. The Dice similarity coefficient was used to assess the U‐Net‐based segmentation performance on both a validation dataset (68 slices) and a test dataset (203 slices). The measurement of cross‐sectional area and Hounsfield unit (HU) density of muscle, VAT and SAT were compared between two methods.

**Results:**

The segmentation for muscle, VAT and SAT demonstrated excellent performance for both the validation (Dice similarity coefficients >0.98, respectively) and test (Dice similarity coefficients >0.97, respectively) datasets. There was a strong positive correlation between manual and AI segmentation measurements of body composition for both datasets (Spearman's correlation coefficients: 0.944–0.999, *P* < 0.001).

**Conclusions:**

Compared to the gold standard, this fully automated segmentation system exhibited a high accuracy for assessing segmentation and quantification of abdominal muscle and adipose tissues of CT slices at the L3 in CRC patients.

## Graphical abstract

The automated analysis of body composition using computed tomography (CT) slices plays a crucial role in understanding its relationship with clinical outcomes in colorectal cancer patients. This study aimed to assess the effectiveness of an AI‐based model for automatic quantification of body composition from lumbar (L3) region CT slices in colorectal cancer patients.

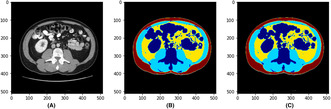

## Introduction

Colorectal cancer (CRC) is the third most common cancer worldwide,^1^ often treated with multimodal therapy.^2^ Body composition measurements, including skeletal muscle (SM), visceral adipose tissue (VAT) and subcutaneous adipose tissue (SAT), have all been shown to be associated with survival‐related clinical outcomes in these patients.^3^, ^4^, ^5^, ^6^, ^7^ In particular, both surgical outcomes, as well as chemotherapy toxicities,^5^ are strongly influenced by patients' body composition.

Computed tomography (CT) is the gold standard for the accurate assessment of body composition;^8^ however, it is currently still a research tool as existing approaches for assessing body composition rely primarily on manual segmentation of CT slices, which is currently a time‐consuming and skill‐intensive procedure.

Deep learning algorithms provide a fundamental technique in artificial intelligence (AI)^9^ and have already been applied to segmentation of body composition in several studies around the world.^10^, ^11^, ^12^, ^13^, ^14^, ^15^ In comparison with manual segmentation, automated segmentation of body composition provided by these algorithms has been shown to be accurate and reliable at the lumber 3 (L3) level.^10^, ^11^, ^12^, ^13^, ^14^, ^15^, ^16^ Previous investigations typically included varied study groups, consisting of individuals with various clinical pathologies and from different backgrounds. Differences in patients and disease‐specific characteristics, along with differences in the protocol and methodologies used for establishing the gold standard, that is manual labelling, can significantly affect the accuracy and relevance of these pre‐existing models when applied to a disease‐specific context such as CRC.^10^, ^11^, ^12^, ^13^, ^14^, ^15^ Adapting these models to such contexts typically necessitates extensive retraining and validation to ensure their accuracy and applicability.

Thus, this study aimed to evaluate the accuracy of our own AI‐generated model for the automated quantification of body composition from L3 CT slices and compare its accuracy to that of skilled manual readings.

## Methods

This study was approved by the Western Health Office for Research (Project QA2020.24_63907). The protocol followed the tenets of the Declaration of Helsinki, and all privacy requirements were met.

All analyses were performed in Python 3.8.8 (Python Software Foundation, Wilmington, DE, USA), Spyder IDE 5.3.1 (International Spyder community) or RStudio (version 2022.2.2.485) (Posit, PBC, Boston, MA, USA).

### AI model development

Segmentation automation was developed using a deep learning algorithm based on the U‐Net architecture.^17^ U‐Net is a convolutional neural network including contracting and expansive pathways that form a ‘U’ shape and is one of the most widely used architectures in deep learning for image segmentation.

**Figure 1:**
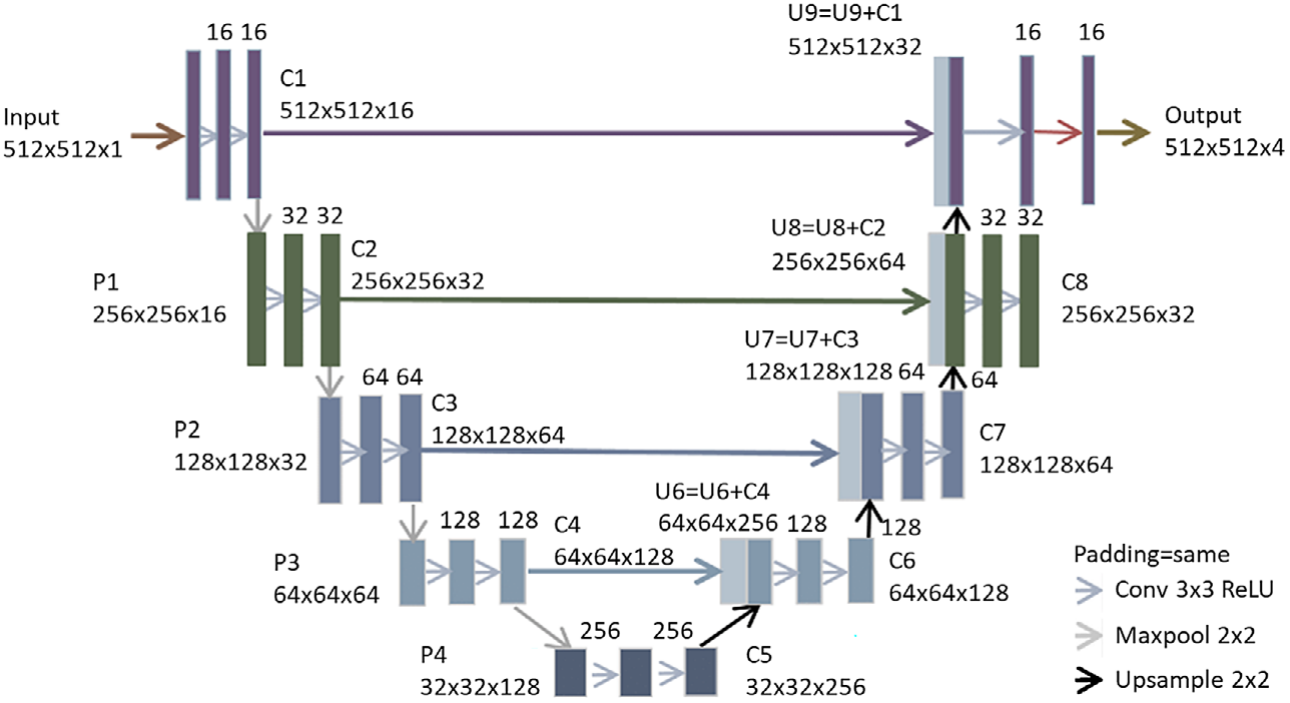
The architecture of U‐Net used in this study.

A two‐dimensional, multiclass U‐Net adapted similarly as indicated by Bhattiprolu^18^, ^19^ was used in this study. Each down‐sampling step consisted of two convolutions (3 3), a ReLU activation function and a max pooling (2 2) operation; the number of convolution filters is doubled from the previous step, beginning with 16 filters in step 1. Each up‐sampling step had a 2 × 2 convolution and two convolutions (3 × 3) with ReLU activation and half the number of filters. In each step, the down‐sampling convolution output was concatenated with the corresponding up‐sampling output. A 0.2 dropout was applied between convolution steps to avoid overfitting. The ‘categorical_cross entropy’ loss function (a loss function for multiclass classification models where there are two or more output labels), a batch size of 1 (equivalent to 512 × 512 pixel) for segmentation and Adam optimization with a learning rate of 0.0005 were used to develop the model. All training, validation and testing were accomplished using Keras (https://keras.io/) and TensorFlow (https://www.tensorflow.org/) using NVIDIA RTX graphics processing unit. The architecture of U‐Net is shown in Figure 1. The appropriate AI model hyperparameters were determined via an internal validation utilising 10% of the training data. The number of epochs used was 200, and the optimal number of epochs selected was 106.

### Study population and CT slices

The AI model was trained and validated using 319 CRC patients recruited from a prospective cancer registry from a single Australian tertiary institution, Western Health. Demographics, including age and gender, were available for 203 patients; the mean age of our cohort was 60.87 ± 12.42, and 52.2% (*n* = 106 patients) were female.

CT slices were collected from scanners from various manufacturers (Philips, Siemens Healthineers, Canon Medical Systems and GE Healthcare). Across all available data, scanners from GE Healthcare were used most often (*n* = 498), followed by those from Canon Medical Systems (*n* = 23) and, finally, others (*n* = 20). CT slices from various manufacturers underwent the same processing procedures. Synapse 5 (FUJIFILM), a medical image viewer which allows clinicians to download and analyse medical images, was used to acquire these CT slices for the analysis. The CT slices taken closest to the colorectal primary surgical resection date were used with a cut‐off being 6 months prior to surgery or 3 months following surgery. Where multiple phase slices were available, supine position and venous phase slices were selected.^20^, ^21^, ^22^ CT slice parameters included whether the slices were contrast‐enhanced with an x‐ray tube voltage of between 100 and 140 kVp. These parameters varied due to settings which were selected based on the clinical requirements.

The axial slices at the L3 vertebral level were selected for the analysis of body composition for each patient. A single slice from the mid‐point of the L3 was manually selected according to Alberta Protocol (https://tomovision.com/Sarcopenia_Help/index.htm). The format of each collected CT slice utilised was the Digital Imaging and Communications in Medicine (DICOM) image, and the image size was 512 × 512 pixels. Hounsfield units (HUs) were used to describe our CT slice pixel values.

### Inclusion and exclusion criteria

Patients were included in the study if they were diagnosed with CRC between 2012 and 2019 at Western Health and had their L3 axial CT slices available. Patients who had suboptimal CT slices, that is with poor visible quality, had evidence of excess amount of SAT that extended outside of the CT slice, had evidence of muscle cut‐off and presented with significant artefacts, secondary to metallic implants, were excluded from the study.

### Training and test datasets

**Figure 2:**
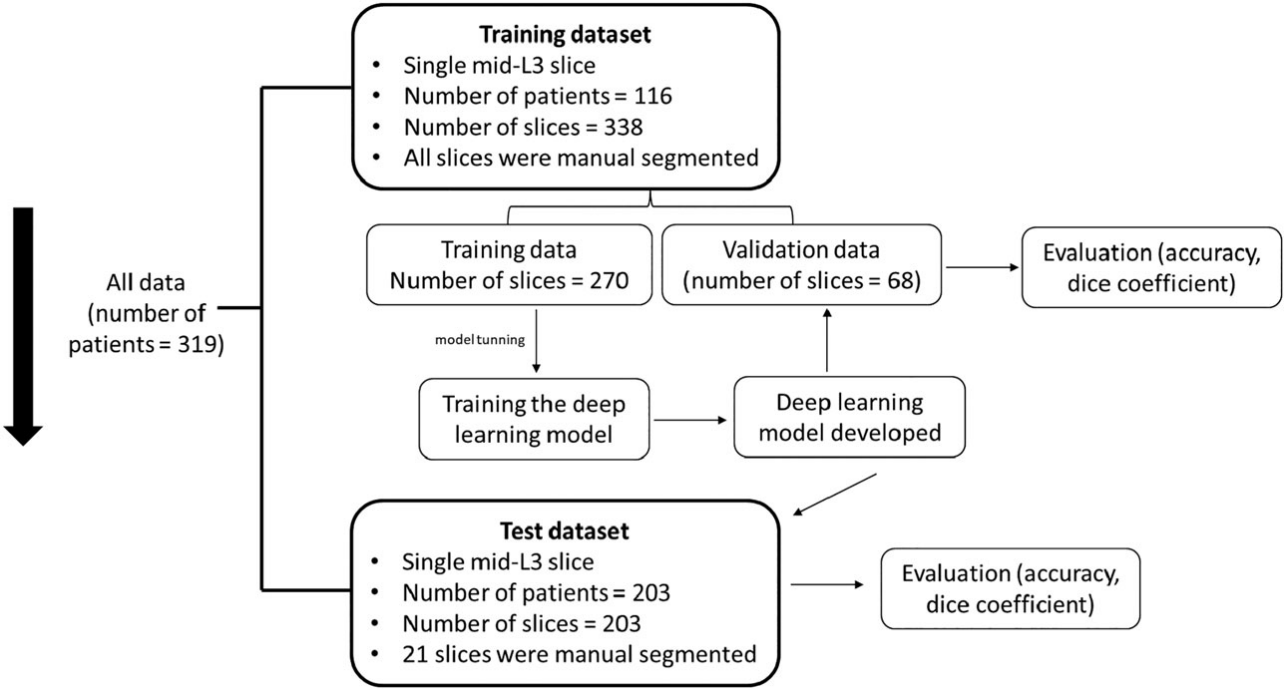
A flow chart demonstrating the model training, validation and testing and the number of CT slices and data sets involved. All patient slices were collected from Western Health.

A flow diagram of how CT slices were used for model training, validation and testing is shown in Figure 2.

The training dataset included 338 CT slices derived from 116 CRC patients. All accessible CT slices (from 6 months prior to surgery or 3 months after surgery) were collected for this dataset, allowing one or more slices to be available for patients. All CT slices of the training dataset were manually segmented. This dataset was then randomly divided into a training dataset (80% of slices, number of slices = 270) and a validation dataset (20% of slices, number of slices = 68). The training dataset was used to build a deep learning model, and the validation dataset was applied to assess the performance of the final fitted model.

To further examine the effectiveness of AI in the quantification of body composition on a single L3 slice, an independent test dataset consisting of 203 CT slices from 203 CRC patients was used. This test dataset was reserved solely for testing and was never included in the model training process prior to testing. Of the CT slices of this test dataset, 1 in 10 slices (number of slices = 21) were randomly selected for ‘gold standard semi‐automated segmentation reading’ for cross‐validation.

### Semi‐automated manual reading

A semi‐automated software (Slice‐O‐Matic version 5.0; TomoVision, Quebec, Canada) was used to segment body composition as our gold standard measurement. The segmentation process was carried out in line with the Alberta Protocol (https://tomovision.com/Sarcopenia_Help/index.htm). Segmentation of the training dataset was performed by experienced graders, both of whom were intensively trained and experienced graders. Prior to segmenting the CT slices for this study, two graders conducted an internal evaluation on 20 slices and reached a consensus agreement. The following values were used as the threshold values for the segmentation tool in line with the Alberta Protocol: SM: −29 to 150 HU, VAT: −150 to −50 HU and SAT: −190 to −30.

### Body composition

SM, VAT and SAT were studied as components of body compositions. The formula used to calculate the area (in cm^2^) was determined by the pixels of a body composition and the pixel spacing. The pixel spacing was derived from the data included within each DICOM file. The radiodensity of a particular body composition was determined by averaging the values of pixels representing that body composition.

### Statistical analysis

The following metrics were used to objectively assess the deep learning model's performance:

- Pixel accuracy: Pixels in the slice that are classified correctly.
- Sørensen–Dice index (Dice coefficient): This presents as 2 × the area of overlap divided by the total number of pixels in both predicted segmentation and the ground truth (semi‐automated reading). The Dice coefficient of each class was calculated, and the average Dice coefficient of the slice was calculated by taking the dice of each class and averaging them:

$$\text{Dice coefficient}=\frac{2\times \text{Area of overlap}}{\text{Total number of pixels in both slices}}$$

The difference between manual and AI‐based measurements of area (in cm^2^) and radiodensity (in HU) of SM, VAT and SAT was compared. The Mann–Whitney test was performed to determine whether there was a statistically significant difference in each measurement between the two groups. The Bland–Altman method was used to measure the agreement between manual reading and segmentation results, and the units of measure used were area (in cm^2^) and radiodensity (in HU) of muscle, VAT and SAT. The mean difference for each measurement was calculated, and the limits of agreement were established, which define the expected range of difference between the two measurement methods. The manual reading with automation segmentation was further compared using Spearman's correlation coefficient (ρ).

## Results

### AI performance in validation dataset

**Figure 3:**
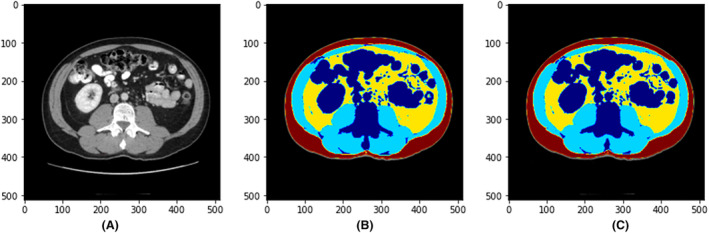
A sample case demonstrating the original CT slice (A), the manual segmented single slice (B) and the AI‐segmented single slice (C).

The pixel accuracy, average Dice coefficient and Dice coefficient for each body composition on the validation dataset in the training process (number of slices = 68) comparing the deep learning model and the manual reading were computed. The average pixel accuracy was 0.99 (0.97–0.99). The average Dice coefficient for all body composition segmentation was 0.98, with 0.98 for muscle, 0.98 for VAT and 0.99 for SAT. Figure 3 shows a sample case demonstrating the original CT slice (A), the manual segmented single slice (B) and the AI‐segmented single slice (C).

The manual and AI‐based measurements of area (in cm^2^) and radiodensity (in HU) of muscle, VAT and SAT were compared and presented in Table 1. There was no significant difference in any of the measurements.

**Table 1:** Semi‐automated manual segmentation and AI‐based segmentations of area (in cm^2^) and radiodensity (in HU) of muscle, VAT and SAT in test datasets.

	Validation dataset (number of slices = 68 slices)		Test dataset (number of slices = 203 slices)	
	Area (in cm^2^)	Radiodensity (in HU)	Area (in cm^2^)	Radiodensity (in HU)
	Mean ± SD			
Muscle				
Manual	136.86 ± 33.74	38.7 ± 10.17	137.33 ± 39.84	36.66 ± 7.54
AI	137.55 ± 33.22	38.66 ± 9.87	138.87 ± 40.98	36.94 ± 7.36
*P* value	0.82	0.98	0.75	0.86
VAT				
Manual	169.47 ± 112.57	−87.82 ± 9.15	206.39 ± 121.44	−92.95 ± 8.13
AI	172.37 ± 113.91	−87.57 ± 9.24	210.5 ± 122.04	−94.36 ± 9.52
*P* value	0.81	0.85	0.78	0.80
SAT				
Manual	200.49 ± 95.29	−98.73 ± 11.15	211.46 ± 97.72	−101.15 ± 8.16
AI	200.96 ± 95.2	−98.96 ± 11.34	212.72 ± 97.49	−101.38 ± 8.64
*P* value	0.95	0.69	0.90	0.88

*P* value was calculated based on the Mann–Whitney test. AI, AI segmentation of CT slices; Manual, manual labelling of CT slices; SAT, subcutaneous adipose tissue; SD, standard deviation; VAT, visceral adipose tissue.

The median of the area (in cm^2^) for muscle, VAT and SAT was 135.34 (interquartile range (IQR): 110.13, 159.36), 155.02 (IQR: 83.51, 250.48) and 182.34 (IQR: 138.15, 256.41) derived from the AI model constructed, respectively. The median absolute errors (in cm^2^) between our model and manual reading for muscle, VAT and SAT on the validation dataset were 1.44 (IQR: 0.94, 2.96), 2.43 (IQR: 0.92, 4.67) and 1.12 (IQR: 0.40, 3.61), respectively.

The median of the radiodensity (in HU) for muscle, VAT and SAT was 38.10 (IQR: 31.68, 46.75), −89.68 (IQR: −93.91, −82.14) and −102.55 (IQR: −106.12, −96.23) derived from the AI model constructed, respectively. The median absolute errors (in HU) between our model and manual reading for muscle, VAT and SAT on the validation dataset were 0.28 (IQR: 0.15, 0.57), 0.54 (IQR: 0.29, 0.91) and 0.28 (IQR: 0.11, 0.60), respectively.

Spearman's correlation coefficient revealed a strong positive correlation between ground truth and segmentation measurements. Spearman's correlation coefficients were given as follows: (A) muscle area: ρ = 0.995 (*P* < 0.001); (B) muscle density: ρ = 0.999 (*P* < 0.001); (C) VAT area: ρ = 0.999 (*P* < 0.001); (D) VAT density: ρ = 0.985 (*P* < 0.001); (E) SAT area: ρ = 0.998 (*P* < 0.001); and (F) SAT density: ρ = 0.985 (*P* < 0.001).

**Figure 4:**
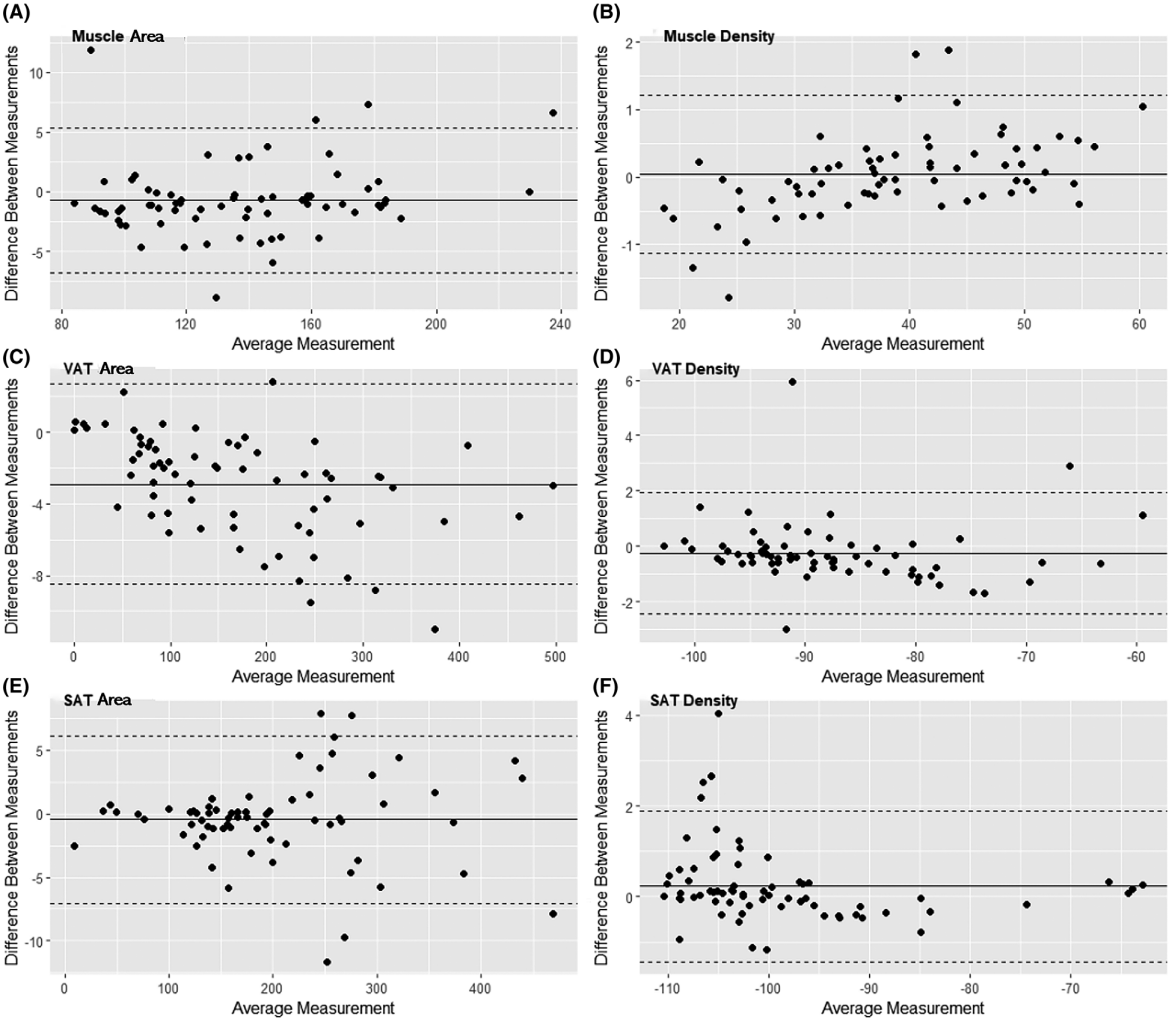
Bland–Altman plots showing differences between AI segmentation and human ground truth for (A) muscle area, (B) muscle HU, (C) VAT area, (D) VAT HU, (E) SAT area and (F) SAT HU. The solid line represents the mean difference, and the dotted line represents the upper and lower limits of agreement. SAT, subcutaneous adipose tissue; VAT, visceral adipose tissue.

Bland–Altman plots comparing the measurements between AI segmentation and manual reading per slice are shown in Figure 4. The mean differences between AI segmentation and manual reading were −0.70 (limits of agreement: −6.75 to 5.36), −2.90 (limits of agreement: −8.48 to 2.68) and −0.47 cm^2^ (limits of agreement: −7.09 to 6.16) for muscle, VAT and SAT areas, and 0.04 (limits of agreement: −1.14 to 1.21), −0.25 (limits of agreement: −2.42 to 1.93) and 0.23 HU (limits of agreement: −1.43 to 1.89) for muscle, VAT and SAT radiodensities, respectively. All Bland–Altman plots showed an even distribution of the differences above and below the mean.

### AI performance in test dataset

The resultant model was further tested using 203 slices in the test dataset, against the measurements obtained from manual reading. The average Dice coefficient for the AI model constructed compared with the semi‐automated manual reading was 0.98, with 0.97 for muscle, 0.98 for VAT and 0.98 for SAT.

The manual and AI‐based measurements of area (in cm^2^) and radiodensity (in HU) of muscle, VAT and SAT are compared in Table 1. There was no significant difference in any of the measurements.

The median of the area (in cm^2^) for muscle, VAT and SAT was 138.74 (IQR: 106.93, 158.82), 184.1 (IQR 147.1, 292.3) and 198.26 (IQR 146.60, 264.87) derived from the AI model constructed, respectively. The median absolute errors (in cm^2^) between our model and manual reading for muscle, VAT and SAT on the test dataset were 1.88 (IQR: 1.39, 2.78), 4.21 (IQR: 1.60, 5.83) and 1.86 (IQR: 1.02, 5.07), respectively.

The median of the radiodensity (in HU) for muscle, VAT and SAT was 38.00 (IQR: 32.06, 40.78), −94.70 (IQR −97.00, −91.93) and −102.64 (IQR −106.91, −98.88) derived from the AI model constructed, respectively. The median absolute errors (in HU) between our model and manual reading for muscle, VAT and SAT on the test dataset were 0.23 (IQR: 0.08, 0.49), 0.40 (IQR: 0.24, 0.69) and 0.29 (IQR: 0.21, 0.71), respectively.

Spearman's correlation coefficients were given as follows: (A) muscle area: ρ = 0.997 (*P* < 0.001); (B) muscle radiodensity: ρ = 0.997 (*P* < 0.001); (C) VAT area: ρ = 0.999 (*P* < 0.001); (D) VAT radiodensity: ρ = 0.978 (*P* < 0.001); (E) SAT area: ρ = 0.999 (*P* < 0.001); and (F) SAT radiodensity: ρ = 0.944 (*P* < 0.001), revealed a strong positive correlation between manual and AI segmentation measurements of body composition.

### Time efficiency

The average time taken for a grader to manually read and annotate one slice was 13.81 min (829.23 sec) (standard deviation (SD): 127.09 sec, number of slices = 78) compared to 0.17 sec (SD: 0.04) for the AI model. The segmentation performed by the AI model was more than 4000 times quicker per slice than that of an experienced human reader.

## Discussion

In this work, an AI model was developed based on a U‐Net deep learning algorithm and validated using patients with CRC treated at a single university hospital of tertiary referral in Melbourne, Australia. When compared to human interpretation, the generated AI model enabled accurate body composition segmentation at the L3 vertebra for muscle, VAT and SAT (average Dice coefficient of 0.98) measurements. The differences between assessments by an experienced human reader and those by the AI‐based segmentation system were small (average less than 5 cm^2^ area and less than 1 HU radiodensity), demonstrating that the AI‐based segmentation system possesses comparable performance in terms of measurement.

Currently, the time‐intensive nature of the manual labelling procedure presents as one of the primary obstacles to employing body composition measurement in routine clinical care. According to our data, manually segmenting a single slice takes an average of 13.81 min by a proficient and experienced grader. Using our AI model, the time to perform this body segmentation analysis was reduced to 0.17 sec, a speed improvement of 4000 times, which appeared to have excellent performance in our datasets. Thus, the time efficiency, accuracy and effectiveness of our AI surpass the performance of the existing manual approach and have the potential to be utilised in the clinical setting.

The majority of our CT slices (*n* = 498) were produced by scanners from GE Healthcare, followed by those from Canon Medical Systems (*n* = 23). In our test dataset (*n* = 21 slices), AI‐based segmentation achieved the same level of performance for GE Healthcare and Canon Medical Systems CT slices, with an average Dice coefficient of 0.98. Due to the fact that 92% of the slices were acquired from the same scanner (GE Healthcare) and only a small number of slices were available from other scanners, we did not train separate AI models for each scanner, but the results demonstrated that our AI model was able to segment CT slices from a range of manufacturers, suggesting that it may be applicable to clinical institutions that use different CT equipment.

### Comparison of other AI segmentation methods at L3 (advantages and limitations)

A number of studies have shown that automated segmentation of abdominal muscle and/or fat yields similar results (Table 2). These include the fully convolutional network (FCN)‐based model developed by Park et al.,^14^ which achieved 0.97 for segmenting muscle VAT and SAT; the U‐Net model developed by Bridge et al.^12^ achieved 0.95–0.98 in segmenting muscle, VAT and SAT, respectively; and the U‐Net model developed by Weston et al.^11^ achieved 0.96, 0.94 and 0.98 in segmenting muscle, VAT and SAT, respectively. Several studies concentrated only on segmenting muscle, these included the FCN model developed by Lee et al.,^10^ which achieved 0.93 Dice similarity for segmenting muscle; the U‐Net model developed by Burns et al.,^13^ which achieved 0.94 Dice similarity for segmenting muscle. Our model's segmentation performance was comparable or superior to other investigators and capable of segmenting all three body compartments of VAT, SAT and muscle simultaneously. For example, the U‐Net model developed by Paris et al.^15^ achieved 0.98, 0.98 and 0.99 in segmenting muscle, VAT and SAT, respectively, with our model achieving similar performance. While direct comparisons of model performances are difficult between different studies, this study wished to indicate that a range of AI‐based models have now been developed and these all achieve similar metrics. These findings indicate a robustness of the methodologies and their applicability in this field. Finally, several research studies^23^ have already begun to segment 3D body composition volume. Future research is required to evaluate the efficacy of the resultant model in segmenting additional slices in order to produce a comprehensive depiction of an individual's body composition.

**Table 2:** Mean Dice coefficients of segmenting muscle, VAT and SAT on single axial L3 slice in previous studies compared to the current model; where performance of both training and test was available, test dataset performance was extracted.

First author (publication year, study design)	Country (Study group)	Sample size	Mean Dice coefficients		
			Muscle	VAT	SAT
Lee et al.^10^ (2017, retrospective)	USA (subjects with lung cancer)	400 CT slices (400 patients)	0.93	‐	‐
Weston et al.^11^ (2018, retrospective)	USA (subjects with pancreatic cancer, renal cell carcinoma, transitional cell carcinoma, or gastrointestinal cancer)	Data 1: 2700 slices (1429 patients)	0.96	0.94	0.98
		Data 2: 2369 slices (1083 patients)	0.92	‐	0.94
Bridge et al.^12^ (2018, retrospective)	USA (subjects with biopsy‐proven pancreatic adenocarcinoma and lymphoma)	1129 CT slices (did not specify)	0.97	0.95	0.98
Burns et al.^13^ (2020, retrospective)	USA (patients age 59 years or older, without regard for patient diagnosis)	102 CT slices (102 patients)	0.94		
Park et al.^14^ (2020, retrospective)	Korea (subjects with gastric cancer, pancreatic cancer, and sepsis and healthy individuals)	1480 CT slices (964 patients)	0.96–0.97	0.97	0.97
Paris et al.^15^ (2020, retrospective)	Canada, the USA, France, and the Netherlands (renal and liver donors, critically ill, liver cirrhosis, pancreatic cancer, and clear cell renal cell cancer patients)	893 slices (893 patients)	0.98	0.98	0.99
Current study (2022, retrospective)	Australia (CRC patients)	541 slices (319 patients)	0.97	0.98	0.98

The studies were sorted by year of publication. SAT, subcutaneous adipose tissue; VAT, visceral adipose tissue.

The major focus of our investigation has been on CRC, whereas the majority of prior studies^11^, ^12^, ^13^, ^14^, ^15^ (Table 2) have featured more varied study groups, comprising patients with varying clinical pathologies and from different backgrounds, thereby providing a broad clinical cohort and range of body composition characteristics. This study is more disease‐specific as the model was developed to learn the characteristics of body composition specifically in CRC patients. It suggested that our model may be more suited than other models for CRC‐related body composition analysis where prior focus was on a range of cancers rather than specifically for CRC. Future studies would be useful to compare various existing models in this specific patient group to assess their effectiveness in segmenting body composition.

All investigations so far utilised a retrospective design, as this is a common constraint in AI research pertaining to the field. The ultimate objective of developing such segmentation tools is to facilitate the use of ‘real‐time’ body composition measures in clinical settings in order to provide tailored ‘personalised’ management plan to the patient in the ‘real world’. In addition, the model developed in this study has not been evaluated on an external dataset (i.e. other clinics or countries). Variations in patient populations and possible variances in CT settings might have an impact on performance. In future investigations, it is anticipated that more internal and external datasets will be acquired and analysed in an effort to evaluate our model with data from various clinics, nations, physicians and settings in order to verify its robustness.

While CT slices with a range of kVp values were included in this study, a consistent distribution of kVp values was maintained across our training, validation and test datasets to ensure the robustness of our model. Most of our patients were scanned at a kVp of 120. Specifically, this kVp value accounted for 93% (252 patients) in the training dataset and 89% (79 patients) in the validation and test datasets. Additionally, 5% (14 patients) in the training dataset and 9% (8 patients) in the validation and test datasets underwent CT scans at a kVp of 100. The model's high performance demonstrated its capability in processing CT slices with different tube voltages effectively. On the other hand, there was a limitation related to the inclusion of more diverse kVp values. The comparatively small number of patients scanned with CT at kVp settings, such as at 110 and 140, may affect the model's capability to fully capture the radiodensity characteristics specific to these kVp settings. This observation highlighted the necessity for future research to include enough patients over a diverse range of kVp values to ensure the applicability of the segmentation model developed in diverse CT settings.

### Future directions

Future research should include plans to investigate other aspects of body composition, such as intramuscular adipose tissue (IMAT), which was also a significant clinical outcome predictor for colorectal cancer patients,^24^ as well as consider pattern or ‘texture’ variations.

One of the challenges clinical applications must face is the integration of the AI model into the clinical process. The present work produced an AI model that could operate alone, as opposed to one that could be integrated. Hospital clinical integration will necessitate future work to assess optimal pipelines and integration into clinical electronic medical record systems.

An exciting recent development is the possibility of using AI body composition analysis to assign accurate dosing of chemotherapy agents in colorectal cancer patients in order to help reduce the significant incidence of chemotherapy‐induced toxicities.^25^, ^26^

## Conclusion

In the current study, we developed and evaluated an AI‐generated model tailored for CRC patients, focusing on automated segmentation and quantification of body composition, based on a single slice at the L3 vertebral level. This provides a more promising way to obtain segmentation values for different body tissues including area and radiodensity of muscle, VAT and SAT, which are crucial in the clinical assessment of CRC patients. The created AI model can automatically and precisely segment and characterise body composition, thereby saving time compared to an experienced human reader when creating a clinical report.

## Conflict of Interest

The authors declare no conflict of interest.

## Data Availability

The data that support the findings of this study are available on request from the corresponding author. The data are not publicly available due to privacy or ethical restrictions.
